# Efficacy of chemotherapy and thermotherapy in elimination of *East African cassava mosaic virus* from Tanzanian cassava landrace

**DOI:** 10.1111/jph.12725

**Published:** 2018-08-12

**Authors:** Christina Edward Kidulile, Elijah Miinda Ateka, Amos Emitati Alakonya, Joseph Canisius Ndunguru

**Affiliations:** ^1^ Department of Biotechnology Mikocheni Agricultural Research Institute Dar es Salaam Tanzania; ^2^ Institute for Biotechnology Research Jomo Kenyatta University of Agriculture and Technology Nairobi Kenya; ^3^ Department of Horticulture Jomo Kenyatta University of Agriculture and Technology Nairobi Kenya; ^4^ International Institute of Tropical Agriculture Ibadan Nigeria

**Keywords:** cassava mosaic begomoviruses, EACMV, ribavirin, salicylic acid, virus‐free plantlets

## Abstract

Cassava mosaic disease is caused by cassava mosaic begomoviruses (CMBs) and can result in crop losses up to 100% in cassava (*Manihot esculenta*) in Tanzania. We investigated the efficacy of chemotherapy and thermotherapy for elimination of *East African cassava mosaic virus* (EACMV) of Tanzanian cassava. In vitro plantlets from EACMV‐infected plants obtained from coastal Tanzania were established in the greenhouse. Leaves were sampled from the plants and tested to confirm the presence of EACMV. Plantlets of plants positive for EACMV were initiated in Murashige and Skoog (MS) medium. On the second subculture, they were subjected into chemical treatment in the medium containing salicylic acid (0, 10, 20, 30 and 40 mg/L) and ribavirin (0, 5, 10, 15 and 20 mg/L). In the second experiment, EACMV‐infected plantlets were subjected to temperatures between 35 and 40°C with 28°C as the control. After 42 days of growth, DNA was extracted from plant leaves and PCR amplification was performed using EACMV specific primers. It was found that plant survival decreased with increasing levels of both salicylic acid and ribavirin concentrations. In general, plants treated with salicylic acid exhibited a lower plant survival % than those treated with ribavirin. However, the percentage of virus‐free plants increased with an increase in the concentration of both ribavirin and salicylic acid. The most effective concentrations were 20 mg/L of ribavirin and 30 mg/L of salicylic acid; these resulted in 85.0% and 88.9% virus‐free plantlets, respectively. With regard to thermotherapy, 35°C resulted in 79.5% virus‐free plantlets compared to 69.5% at 40°C. Based on virus elimination, ribavirin at 20 mg/L, salicylic acid 30 mg/L and thermotherapy at 35°C are recommended for production of EACMV free cassava plantlets from infected cassava landraces.

## 1 INTRODUCTION

Cassava (*Manihot esculenta*) is a major staple food crop in sub‐Saharan Africa (SSA) where it accounts for 176 million metric tons of the 270 million metric tons of annual global production (FAO, 2014). Despite its important role in alleviating food security, there has been a low productivity of cassava per unit area in SSA (Nweke, Spencer, & Lynam, 2002). This has mainly occurred due to the impact of viral diseases such as cassava mosaic disease (CMD) (Bull, Ndunguru, Gruissem, Beeching, & Vanderschuren, 2011). CMD is caused by at least nine species of cassava mosaic begomoviruses (CMBs) that can be broadly categorized as African cassava mosaic viruses (ACMVs) and East African cassava mosaic viruses (EACMVs). These viruses are transmitted by whiteflies (*Bemisia tabaci* Gennadius) and spread through infected planting materials and contaminated implements, and their existence can cause yield losses of up to 100% on susceptible genotypes (Varma & Malathi, 2003).

Virus elimination is a technique used for the production of virus‐free planting materials. There are several virus elimination methods, including thermotherapy, chemotherapy, electrotherapy, somatic embryogenesis and meristem tip culture (Panattoni, Luvisi, & Triolo, 2013). These methods can be used alone or in combination with other methods to obtain virus‐free plantlets (Paprstein et al., 2008).

There have been reports of successful elimination of viruses from infected plants through chemotherapy and thermotherapy, for example Hu et al. (2015). In another study, chemotherapy produced 100% virus‐free grapevine plants with simple and mixed infection (Guta, Buciumeanu, Gheorghe, & Al, 2014). Furthermore, the elimination of Cassava brown streak virus from cassava was achieved by combining chemotherapy and thermotherapy in Kenya (Mwagangi, Ateka, Nyende, & Kagungu, 2014). However, currently efficacy of chemotherapy and thermotherapy in eliminating viruses causing CMD from Tanzanian landraces is unknown. The aim of this study was to evaluate the effectiveness of chemotherapy and thermotherapy in eliminating EACMV in widely grown, farmer‐preferred cassava landraces in Tanzania.

## 2 MATERIALS AND METHODS

### 2.1 Source of plants and screening for the presence of cassava mosaic begomoviruses

Twenty stem cuttings of the cassava cultivar Kibandameno showing CMD symptoms were collected from Tanga region in Tanzania, established and maintained in a greenhouse at Mikocheni Agricultural Research Institute. Ten days after sprouting, leaves were collected and used for DNA extraction using the cetyl trimethyl ammonium bromide (CTAB) method (Lodhi, Ye, Weeden, & Reisch, 1994; Xu, Aileni, Abbagani, & Zhang, 2010).

The extracted DNA was subjected to polymerase chain reaction using the primer pair EAB555, forward (5′‐TACATCGGCCTTTGAGTCGCATGG‐3′) and reverse (5′‐ CTTATTAACGCCT ATATAAACACC‐3′) that amplifies approximately 555 bp nucleotides. The PCR reaction mix contained 17.3 μl of sterile distilled water, 1 μl dNTPs (2.5 mM), 2.5 μl ×10 PCR buffer + 20 mM MgCl_2_, 0.2 μl Taq polymerase, 1.0 μl of primer mix and 2 μl of DNA template. PCR conditions were as follows; predenaturation at 94°C for 4 min, followed by 35 cycles of denaturation at 94°C for 1 min, annealing at 56°C for 1 min and elongation at 72°C for 2 min and final elongation at 72°C for 10 min. Samples were then stored at 4°C until used for further analysis. PCR amplification was checked by loading 5 μl of PCR products in 1.5% agarose gel stained with ethidium bromide submerged in 1X TE buffer for 50 min at 180 V. The PCR products were visualized on a gel documentation system (BioDoc‐It^®^ 210, USA) and photographed.

### 2.2 Multiplication of EACMV positive cassava plants

Murashige and Skoog medium (MS) supplemented with 20 g/L sucrose and 3 g/L plant agar was sterilized at 121°C for 15 min before being used for initiation and multiplication of EACMV‐infected cassava plants.

Nodal cuttings of EACMV‐infected cassava plants were taken using a clean surgical blade and samples were washed vigorously with tap water containing detergent (Tarmol^®^) and three drops of Tween‐20 to achieve surface sterilization. The explant was then taken to a safety hood and washed with 70% ethanol for 5 min. They were subsequently rinsed in sterile distilled water three times. The explants were then soaked in sodium hypochlorite containing Tween‐20 for 2 min and then rinsed with sterile distilled water until foam had been removed. The ends of the nodes were cut with a sterile surgical blade, grown on MS medium and incubated in a growth room at 28°C with a photoperiod of 16 hr light and 8 hr dark. Subculturing was performed after 5 weeks.

### 2.3 Virus elimination

#### 2.3.1 Chemotherapy

Salicylic acid and ribavirin were used as antiviral compounds. They were prepared and filter‐sterilized using a 0.22 μm Millipore filters. Nodal cuttings from infected cassava were grown in MS medium supplemented with salicylic acid at concentrations of 0, 10, 20, 30 and 40 mg/L and ribavirin at concentrations of 0, 5, 10, 15 and 20 mg/L for three weeks at 28°C in a photoperiod of 16 hr light and 8 hrr dark. After 3 weeks the plants were transferred into medium without antiviral compounds before PCR analysis was performed.

#### 2.3.2 Thermotherapy

Infected nodal cuttings from subcultured plants were grown in MS media in a tissue culture growth room at 28°C for 2 weeks and then moved to a heat chamber with 16 hr light and 8 hr dark for 3 weeks. Temperature levels were set at 28°C (control), 30°C, 35°C and 40°C.

### 2.4 Experimental design, data collection and analysis

The chemotherapy experiment had two treatments, composed of salicylic acid and ribavirin at five concentration levels for each. Each level of treatment contained 12 plants, replicated four times making a total of 480 plants for the chemotherapy experiment. The thermotherapy experiment had four levels of temperature, 28, 30, 35 and 40°C. At each level of temperature, 12 plants were used, replicated four times. Surviving plants were recorded, virus‐ tested and EACMV elimination expressed as percentage virus‐free plants out of those survived. Data were analysed by analysis of variance (ANOVA) with a 5% level of significance, using Genstat (Lawes Agricultural Trust, Rothamsted Experimental Station 2006, Version 15).

## 3 RESULTS

**Figure 1:**
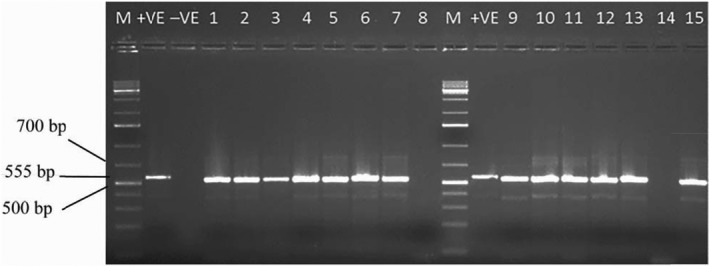
Detection of EACMV‐infected cassava. Lane 1, 1 kb molecular marker; lane 2 and lane 3, are positive and negative control, respectively. Lane numbers 1 to 15 correspond to the infected cassava plants that were tested

The PCR products from tested plants were visualized in agarose gel. Of the leaf samples tested, 13 gave PCR products using EACMV‐ specific primers while 2 did not amplify (Figure 1). The EACMV positive plants were cultured and used for thermotherapy and chemotherapy treatment.

### 3.1 Effect of ribavirin on survival and growth of in vitro cassava plants

**Figure 2:**
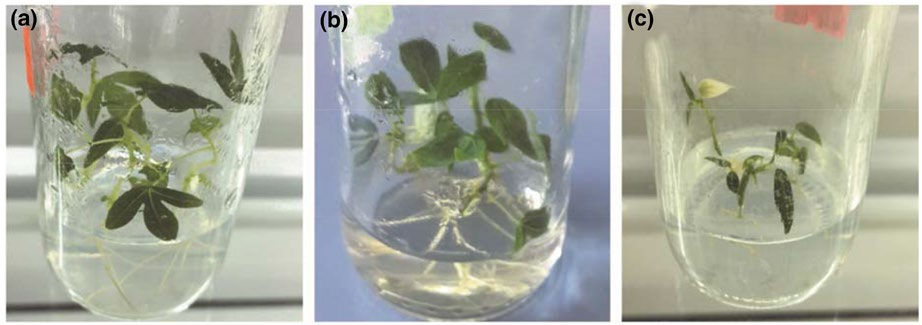
Growth of in vitro cassava plantlets on Murashige and Skoog (MS) medium after 42 days on ribavirin treatment. Control 0 mg/L (a), 10 mg/L (b), 20 mg/L (c)

A total of 123 out of 192 cassava plants survived treatments with ribavirin at different concentrations (Table 1). There was a significant difference (*p *≤* *0.001) between different concentrations (Appendix 1.i) on survival % and virus‐free plants. After 42 days of culture some plant leaves and stems at concentration of 20 mg/L became chlorotic (Figure 2c) on ribavirin‐treated plants and growth was reduced. This was due to the phytotoxic effects of the chemicals such as leaf necrosis and chlorosis, which also led to leaf defoliation. Plants treated with medium concentration (10 mg/L) showed normal growth (Figure 2b). Control plants exhibited better growth and remained green (Figure 2a). The concentration of antiviral stimulated the number of plants that were regenerated. It was observed that the increase in antiviral concentration decreased the survival percentage of plants as observed in Table 1.

**Table 1:** Means of survival and virus elimination in cassava plants (after 42 days) on ribavirin treatment

Ribavirin conc (mg/L)	Plants initiated	Plants survived	Survival (%)	Survival means	Negative plants (%)*	Negative means	Positive plants (%)*	Positive means
0	48	48	100	3.0d	0 (0.0)	0.0a	48 (100.0)	3.0c
5	48	40	83.3	2.5 cd	21 (52.5)	1.4b	19 (47.5)	1.1b
10	48	35	72.9	2.2bc	23 (65.7)	1.9b	12 (34.3)	0.4a
15	48	28	58.3	1.8ab	20 (71.5)	1.5b	8 (28.6)	0.3a
20	48	20	41.6	1.3a	17 (85.0)	1.1b	3 (15.0)	0.2a
Grand mean				2.1		1.2		1.0
L.s.d				0.52		0.6		0.4
Cv%				35.2		75.9		58.4

*Values in parentheses indicate the percentage of plants tested negative and positive. Means followed by a common letter are not significantly different at *p* ≤ 0.05.

### 3.2 Effect of salicylic acid on survival and growth of in vitro cassava plants

**Figure 3:**
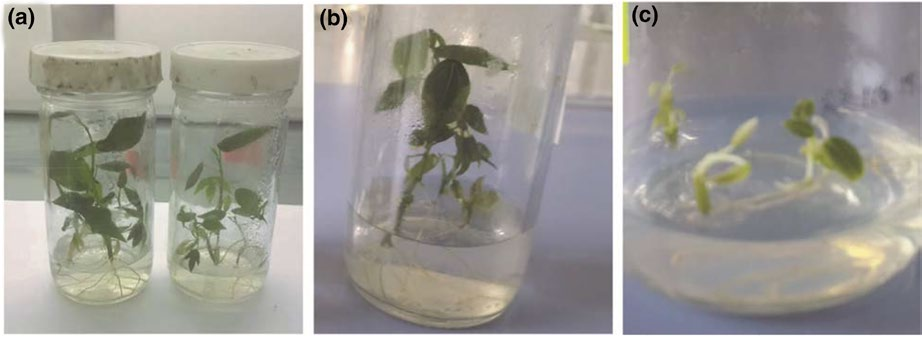
Growth of in vitro cassava plantlets on Murashige and Skoog (MS) medium after 42 days on salicylic acid treatment. Control 0 mg/L (a), 20 mg/L (b), 30 mg/L (c)

Cassava plants surviving treatments with salicylic acid at different concentrations were 69 out of 192 (Table 2 **).** Phytotoxic effects like leaf necrosis and chlorosis was observed on plants treated with salicylic acid at 30 and 40 mg/L after 42 days of culture (Figure 3c) which led to growth reduction of the plantlets, while control plants exhibited normal growth (Figure 3a). There was a significant difference (*p* ≤ 0.001) between different concentrations (Appendix 1.ii) on survival % and virus‐free plants. The number of plants regenerated was proportional to the concentration of salicylic acid. The number of regenerated plants from the salicylic acid treatment at 10 mg/L and 30 mg/L were 32 and 9 respectively. It was observed that the increase in antiviral concentration decreased the survival % of plants. Plants did not survive the highest concentration of salicylic acid (Table 2).

**Table 2:** Means of survival and virus elimination in cassava plants (after 42 days) salicylic acid treatment

Salicylic acid conc (mg/L)	Plants initiated	Plants survived	Survival (%)	Survival means	Negative plants (%)*	Negative means	Positive plants (%)*	Positive means
0	48	48	100	3.0c	0 (0.0)	0.0a	48 (100.0)	3.0c
10	48	32	66.7	1.8b	22 (68.8)	1.1b	10 (31.2)	0.6b
20	48	28	28.3	1.6b	21 (75.0)	1.2b	7 (25.0)	0.3ab
30	48	9	18.8	0.6a	8 (88.9)	0.6ab	1 (11.1)	0.0a
40	48	0	0	0.0a	0 (0.0)	0.0a	0 (0.0)	0.0a
Grand mean				1.4		0.6		0.8
L.s.d				0.5		0.5		0.3
Cv%				49.6		117.3		53.2

*Values in parentheses indicate the percentage of plants tested negative and positive. Means followed by a common letter are not significantly different at *p* ≤ 0.05.

### 3.3 Effect of thermotherapy on growth and survival of in vitro cassava plants

**Figure 4:**
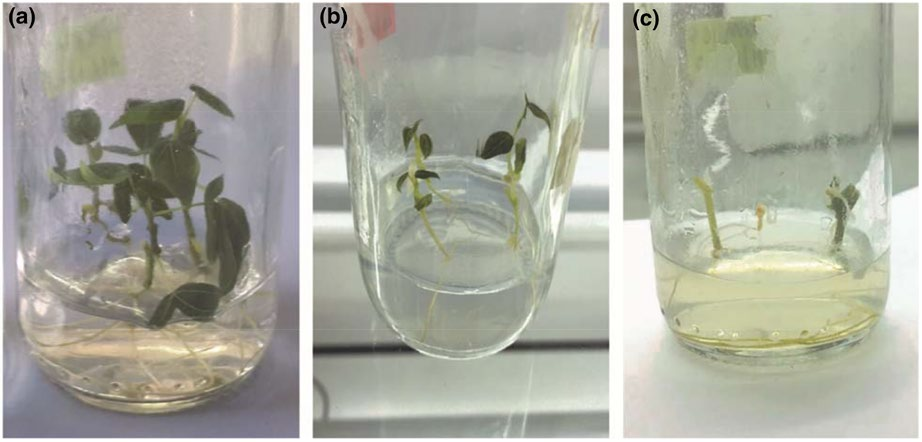
Growth of in vitro cassava plants after 42 days temperature treatment. Control 28°C (a), 35°C (b), 40°C (c)

Plants that were subjected to different temperature regimes had different responses. Results indicate that there was significant difference (*p* ≤ 0.001) between temperature regimes on survival % and virus‐free plants (Appendix 1.iii). All the plants exposed at 28°C grew normally (Figure 4a) while all the plants at higher temperatures (35–40°C) exhibited abnormal growth. Plants subjected at 30°C exhibited normal growth as plants subjected at 28°C. It was observed that at higher temperatures the plant leaves became chlorotic, dried up and dropped off (Figure 4c). Survival % among the four temperature regimes was over 47% (Table 3). Thermotherapy treated plants at higher temperature resulted in lower survival % of 47.9% at 40°C compared to 93.8% at 30°C. Survival decreased with increase in temperature **(**Table 3).

**Table 3:** Means of survival and virus elimination in cassava plants (after 42 days) on thermotherapy

Temperature (°C)	Plants initiated	Plants survived	Survival (%)	Survival means	Negative plants (%)*	Negative means	Positive plants (%)*	Positive means
28	48	48	100	3.0c	0 (0.0)	0.0a	48 (100)	3.0c
30	48	45	93.8	2.5bc	22 (48.9)	0.94b	23 (51.1)	1.6b
35	48	39	81.3	2.3b	31 (79.5)	1.7b	8 (20.1)	0.6a
40	48	23	47.9	1.3a	16 (69.5)	1.0b	7 (30.5)	0.3a
Grand mean				2.3		0.91		1.3
L.s.d				0.5		0.6		0.5
Cv%				32.5		94.9		53.4

*Values in parentheses indicate the percentage of plants tested negative and positive. Means followed by a common letter are not significantly different at *p* ≤ 0.05.

### 3.4 Efficiency of chemotherapy and thermotherapy in elimination of EACMV from infected cassava plants

The results obtained showed that virus elimination efficiency relied on chemical concentration and temperature regime. Higher concentrations and higher temperatures resulted in greater levels of virus elimination. In the 20 mg/L ribavirin treatment, 85% of plantlets were virus‐free, while at 5 mg/L, this figure was 52% (Table 1). For salicylic acid at 30 mg/L, 88.9% plantlets were virus‐free, compared to concentration of 10 mg/L (68% virus‐free) (Tables 2). Thermotherapy resulted in more than 50% virus‐free plants with 35°C showing the highest virus elimination of 79.5%. At 40°C, the number of surviving plantlets was observed to be low but the efficiency of virus elimination was high (69.5%) compared to 30°C which showed 48.9% (Table 3).

## 4 DISCUSSION

The effectiveness of different in vitro techniques for elimination of EACMV from infected cassava plants was assessed. Salicylic acid and ribavirin had different effects on survival and growth of cassava plants. It was observed that plant growth was attenuated at higher concentrations of the antiviral compounds and the leaves turned yellowish and eventually dropped off. This observation is consistent with the study of Hu et al. (2015) who observed slower growth of apple plants treated with ribavirin. However, plants subjected to low concentrations of chemicals in the current study grew better and no defoliation was observed.

The efficiency of virus elimination following chemical treatment was influenced by concentration levels. The rate of virus elimination increased at higher concentrations of both chemicals. At higher concentrations of both antiviral compounds phytotoxic effects were manifested by the low survival % on both experimental variants, where by high mortality of the shoots and defoliation was observed. It was reported by Cieslinska (2007) that while increased concentration of ribavirin increases the effectiveness of virus elimination, it increases phytotoxicity and slows plantlet growth. In the current study, we observed the above findings at 20 mg/L ribavirin treatment. Thus, the higher concentrations required during chemical treatment to inhibit virus multiplication approach toxic levels for plants as observed by Nascimiento, Pio Ribeiro, Willadino, and Andrade (2003). Therefore, a higher number of regenerated clean plants as well as low survival % were found for both ribavirin and salicylic acid‐treated plants at high concentrations (Table 1 and 2). At this point viral RNA mutation exceeds a critical threshold and leads to infectivity and/or extinction of the virus population (Panattoni et al., 2013). On the other hand, a high plant regeneration rate occurred at low concentrations since these levels were less toxic and furthermore, over 50% were virus‐free. These results showed that even at low concentrations virus replication was hindered as in the cases of ribavirin at 5 mg/L and salicylic acid 10 mg/L (Table 1 and 2). Similar results were obtained by Seker, Süzerer, Elibuyuk, and Çiftçi (2015) in eliminating Plum pox potyvirus (PPV) from infected apricot shoots using chemotherapy. Even though salicylic acid‐treated plants had a lower survival % at higher concentration of the chemical, treatment resulted in a greater percentage of virus‐free plants. This result is consistent with that of Mwagangi et al. (2014) where they note a similar effect after chemical treatment.

Regarding temperature, different regimes affected the efficiency of virus elimination from infected cassava plants. Thermotherapy has been widely used to eliminate viruses from trees, herbaceous plants and other vegetative plants (Panattoni et al., 2013; Tan, Wang, Hong, & Wang, 2010; Valero, Ibanez, & Morte, 2003). In recent studies addressing the use of RNA to destroy viruses no relationship was reported between RNA silencing and temperature. Plant‐virus interaction is affected by temperature and higher temperatures are associated with minimal virus levels in infected plants (Qu et al., 2005). Wang, Cuellar, Rajamäki, Hirata, and Valkonen (2008) reported that exposure of plants to heat stress reduces movement of virus particles into the apical meristem by inhibiting viral RNA synthesis. In this study cassava plantlets showed a varying heat treatment response, which ranged from vitrification to total death of the plantlet due to heat stress. The growth rate of temperature treated plants decreased with increasing temperature. At higher temperatures, the rate of virus elimination was greater than at low temperature. However, low plantlet survival was observed at high temperatures (40°C) and higher survival % at low temperatures. Optimal survival was observed with incubation at 35°C for 4 weeks which achieved 79.5% virus‐free plants. This agrees with the findings of Delgado and Rojas (1992) and Acheremu, Akromah, Yussif Jnr, and Santo (2015). The effect of temperature was due to the ability of heat to inhibit virus multiplication (Manganaris, Economou, Boubourakas, & Katis, 2003). The study observed that thermotherapy and chemotherapy eliminated EACMV, with virus‐free plants most optimally obtained at 10 mg/L ribavirin (72.9% survival, 65.7% virus‐free), 10 mg/L salicylic acid (66.7% survival, 68.8% virus‐free) and 35°C (81.2% survival, 79.5% virus‐free).

## 5 CONCLUSION

This study demonstrated that virus‐free plantlets can be produced in vitro from diseased plants thereby enhancing clean seed multiplication and conservation of valuable germplasm. Based on virus‐free percentage, ribavirin at 20 mg/L, salicylic acid 30 mg/L and thermotherapy at 35°C are recommended for production of EACMV free cassava plantlets from infected cassava landraces. Further studies should be conducted to evaluate the combined effects of chemotherapy and thermotherapy on EACMV elimination.

## CONFLICT OF INTEREST

The authors declare that the research was conducted in the absence of any commercial or financial relationships that could be constructed as potential conflict of interest.

## APPENDIX 1 Analysis of Variance (ANOVA) for ribavirin, Salicylic acid and Temperature

### 1.1 Ribavirin

Variate: Survival

**Table:**

Source of variance	d.f	m.s	Fpr
conc	4	7.26	<0.001
Residual	75	0.57	
Total	79		

Variate: Negative

**Table:**

Source of variance	d.f	m.s	Fpr
conc	4	8.48	<0.001
Residual	75	0.80	
Total	79		

Variate: Positive

**Table:**

Source of variance	d.f	m.s	Fpr
conc	4	22.51	<0.001
Residual	75	0.33	
Total	79		

### 1.2 Salicylic Acid

Variate: Survival

**Table:**

Source of variance	d.f	m.s	Fpr
conc	4	21.47	<0.001
Residual	75	0.47	
Total	79		

Variate: Negative

**Table:**

Source of variance	d.f	m.s	Fpr
conc	4	5.36	<0.001
Residual	75	0.46	
Total	79		

Variate: Positive

**Table:**

Source of variance	d.f	m.s	Fpr
conc	4	25.56	<0.001
Residual	75	0.18	
Total	79		

### 1.3 Temperature

Variate: Survival

**Table:**

Source of variance	d.f	m.s	Fpr
conc	3	8.67	<0.001
Residual	60	0.53	
Total	63		

Variate: Negative

**Table:**

Source of variance	d.f	m.s	Fpr
conc	3	7.69	<0.001
Residual	60	0.74	
Total	63		

Variate: Positive

**Table:**

Source of variance	d.f	m.s	Fpr
conc	3	24.25	<0.001
Residual	60	0.51	
Total	63		

## References

[jph12725-bib-0001] Acheremu, K ., Akromah, R ., Yussif Jnr, I ., & Santo, K. G . (2015). Effect of thermotherapy in the elimination of viruses on four (4) mosaic diseased cassava cultivars. Journal of Biology, Agriculture and Healthcare, 4(22), 9–15.

[jph12725-bib-0002] Bull, S. E. , Ndunguru, J. , Gruissem, W. , Beeching, J. R. , & Vanderschuren, H. (2011). Cassava: Constraint to production and the transfer of biotechnology of African laboratories. Plant Cell Reports, 30, 779–787. 10.1007/s00299-010-0986-6 21212961

[jph12725-bib-0003] Cieslinska, M. (2007). Application of thermotherapy and chemotherapy *in vitro* for eliminating some viruses infecting Prunus sp. Fruit trees. Journal of Fruit and Ornamental Plant Research, 15, 117–124.

[jph12725-bib-0004] Delgado, G. E. , & Rojas, C. (1992) Cassava seed production program by meristem culture in unprg lambayeque (Peru). Proceedings of the First International Science Meetings, CBN Cartagena de Indias, Colombia. 25‐28 August 146‐148

[jph12725-bib-0005] FAO . (2014) http://faostat3.fao.org/compare/E

[jph12725-bib-0006] Guta, I. C. , Buciumeanu, E. C. , Gheorghe, R. N. , & Teodorescu, A. L (2014). Elimination of grapevine fleck virus by in vitro chemotherapy. Notulae Botanicae Horti Agrobotanici Cluj‐Napoca, 42(1), 115–118.

[jph12725-bib-0007] Hu, G. , Dong, Y. , Zhang, Z. , Fan, X. , Ren, F. , & Jun, Z. (2015). Virus elimination from in vitro apple by thermotherapy combined with chemotherapy. Plant Cell, Tissue and Organ Culture, 121, 435–443. 10.1007/s11240-015-0714-6

[jph12725-bib-0008] Lodhi, M. A. , Ye, G. N. , Weeden, N. F. , & Reisch, B. (1994). A simple and efficient method for DNA extraction from grapevine cultivars and Vitis species. Plant Molecular Biology Reporter, 12, 6–13. 10.1007/BF02668658

[jph12725-bib-0009] Manganaris, G. A. , Economou, A. S. , Boubourakas, I. N. , & Katis, N. I. (2003). Elimination of PPV and PNRSV through thermotherapy and meristem‐tip culture in nectarine. Plant Cell Reports, 22, 195–200. 10.1007/s00299-003-0681-y 12898177

[jph12725-bib-0010] Mwagangi, M , Ateka, E , Nyende, A , & Kagungu, A. (2014). limination of cassava brown streak virus from infected cassava. Journal of Biology, Agriculture and Healthcare, 4(13), 34–40.

[jph12725-bib-0011] Nascimiento, L. C. , Pio Ribeiro, G. , Willadino, L. , & Andrade, G. P. (2003). Stock indexing and potato virus Y elimination from potato plants cultivated *in vitro* . Scientia Agricola, 60, 525–530. 10.1590/S0103-90162003000300017

[jph12725-bib-0012] Nweke, F. I. , Spencer, D. S. C. , & Lynam, J. K. (2002). The Cassava transformation: Africa's best‐kept secret (pp. 1–7). East Lansing, MI: Michigan State University Press.

[jph12725-bib-0013] Panattoni, A. , Luvisi, A. , & Triolo, E. (2013). Review: Elimination of viruses in plants: Twenty years of progress. Spanish Journal of Agricultural Research, 11, 173–188. 10.5424/sjar/2013111-3201

[jph12725-bib-0014] Paprstein, F. , Sedlak, J. , Polak, J. , Svobodova, L. , Hassan, M. , & Bryxiova, M. (2008). Results of *in vitro* thermotherapy of apple cultivars. Plant Cell, Tissue and Organ Culture, 94(3), 347–352. 10.1007/s11240-008-9342-8

[jph12725-bib-0015] Qu, F. , Ye, X. , Hou, G. , Sato, S. , Clemente, T. E. , & Morris, T. J. (2005). RDR6 has a broad‐spectrum but temperature‐dependent antiviral defense role in Nicotiana benthamiana. Journal of Virology, 79(24), 15209–15217. 10.1128/JVI.79.24.15209-15217.2005 16306592 PMC1316014

[jph12725-bib-0016] Seker, M. G. , Süzerer, V. , Elibuyuk, I. O. , & Çiftçi, Y. Ö. (2015). In vitro elimination of PPV from infected apricot shoot tips via chemotherapy and cryotherapy. International Journal of Agriculture and Biology, 17, 1065–1070.

[jph12725-bib-0017] Tan, R. R. , Wang, L. P. , Hong, N. , & Wang, G. P. (2010). Enhanced efficiency of virus eradication following thermotherapy of shoot‐tip cultures of pear. Plant Cell, Tissue and Organ Culture, 101, 229–235. 10.1007/s11240-010-9681-0

[jph12725-bib-0018] Valero, M. , Ibanez, A. , & Morte, A. (2003). Effects of high vineyard temperatures on the Grapevine leaf roll associated virus elimination from Vitis vinifera L. cv. Napoleon tissue cultures. Scientia Horticulturae, 97, 289–296. 10.1016/S0304-4238(02)00212-1

[jph12725-bib-0019] Varma, A. , & Malathi, V. G. (2003). Emerging geminivirus problems: A serious threat to crop production. Annals of Applied Biology, 142, 145–164. 10.1111/j.1744-7348.2003.tb00240.x

[jph12725-bib-0020] Wang, Q. , Cuellar, W. J. , Rajamäki, M. L. , Hirata, Y. , & Valkonen, J. P. (2008). Combined thermotherapy and cryotherapy for efficient virus eradication: Relation of virus distribution, subcellular changes, cell survival and viral RNA degradation in shoot tips. Molecular Plant Pathology, 8, 1–14. 10.1111/j.1364-3703.2007.00456.x PMC6640318 18705855

[jph12725-bib-0021] Xu, J. , Aileni, M. , Abbagani, S. , & Zhang, P. (2010). A reliable and efficient method for total RNA isolation from various members of sprunge family (Euphorbiaceae). Phytochemical Analysis, 21(5), 395–398. 10.1002/pca.1205 20135710

